# Crocetin exploits p53-induced death domain (PIDD) and FAS-associated death domain (FADD) proteins to induce apoptosis in colorectal cancer

**DOI:** 10.1038/srep32979

**Published:** 2016-09-13

**Authors:** Pallab Ray, Deblina Guha, Juni Chakraborty, Shuvomoy Banerjee, Arghya Adhikary, Samik Chakraborty, Tanya Das, Gaurisankar Sa

**Affiliations:** 1 Division of Molecular Medicine, Bose Institute, P-1/12, CIT Scheme VII M, Kolkata 700 054, India

## Abstract

Tumor suppressor p53 preserves the genomic integrity by restricting anomaly at the gene level. The hotspots for mutation in half of all colon cancers reside in p53. Hence, in a p53-mutated cellular milieu targeting cancer cells may be achievable by targeting the paralogue(s) of p53. Here we have shown the effectiveness of crocetin, a dietary component, in inducing apoptosis of colon cancer cells with varying p53 status. In wild-type p53-expressing cancer cells, p53 in one hand transactivates BAX and in parallel up-regulates p53-induced death domain protein (PIDD) that in turn cleaves and activates BID through caspase-2. Both BAX and t-BID converge at mitochondria to alter the transmembrane potential thereby leading to caspase-9 and caspase-3-mediated apoptosis. In contrast, in functional p53-impaired cells, this phytochemical exploits p53-paralogue p73, which up-regulates FAS to cleave BID through FAS-FADD-caspase-8-pathway. These findings not only underline the phenomenon of functional switch-over from p53 to p73 in p53-impaired condition, but also validate p73 as a promising and potential target for cancer therapy in absence of functional p53.

Colorectal cancer being the third most common form of cancer in the world, accounts for more than 9% of all cancer^1^. Colon carcinogenesis is often a result of accumulation of several genetic and genomic alterations in cells, which consequently lead to cellular proliferation and tumor formation. One of the major events behind such genetic aberration is the inactivation of the tumor suppressor gene p53. p53, among the most commonly mutated genes in all human cancers is associated with an unfavorable prognosis of tumor progression, tolerance to the genomic instability and resistance to apoptosis^2^. Among the well-known functions of p53 mostly highlighted are controlling cell cycle checkpoints and triggering apoptosis in cells upon receiving cellular stress^3^.

About 50% of all colon cancer harbors non‐functional p53 protein as a result of p53 mutations^4^. In fact, many chemotherapeutic agents have failed to show impressive results in cancer with loss of function of p53^2^. In this regard, p73, a p53 family member sharing considerable homology with it, has been shown to function in a manner analogous to p53 by controlling cell cycle checkpoints and DNA damage-induced apoptosis through trans-activation of an overlapping set of p53/p73 target genes^5^. Hence, the idea of certain cellular responses which seemed to be “p53-independent,” might be mediated by this relative of p53. Interestingly, p73 is expressed as two N-terminally distinct isoforms, transcriptionally active TAp73 and transcriptionally inactive ∆Np73^6^. ΔNp73 is dominant-negative to its wild-type counterpart which inhibits TAp73 and is associated with tumor development^7^. ΔNp73 is also frequently over-expressed in a variety of human cancers^8^ but is barely detectable in normal tissues. ΔNp73 efficiently counteracts trans‐activation; apoptosis and growth suppression mediated by wild‐type p53 and TAp73 and also confers drug-resistance to wild‐type p53‐harboring tumor cells^9^.

Various studies have proved that induction of apoptosis is an essential event for therapeutic targeting of cancer cells. Classical pathway of p53-dependent apoptosis exploits BAX-mediated release of cytochrome-c and AIF, which are actively involved in caspase activation and protein or DNA degradation^10^. Besides this, another target of p53 is PIDD (p53-induced death domain protein), which is a well-known regulator of genotoxic stress-induced apoptosis. It achieves its function by forming a multi-protein complex PIDDosome, along with an adaptor protein RAIDD (receptor-interacting protein (RIP)-associated ICH-1/CED-3 homologous protein with a death domain) and caspase-2^11^. Caspase-2 is one of the first and most well conserved mammalian caspase to be identified^12^. The well-versed pro-apoptotic role of caspase-2 in BID cleavage and activation has been well documented^13^,^14^. The death domain of PIDD has been shown to interact with RAIDD, which in turn binds caspase-2 through the caspase-recruitment domain (CARD)^15^. The formation of PIDDosome is required for p53-induced apoptosis^11^,^15^,^16^.

It is well established that apoptosis triggered by extracellular signals activate death receptor family which is different from intrinsic apoptotic signals such as DNA damage, oxidative stress etc.^17^. Extrinsic apoptosis is stimulated by specific ligands such as TNFα, FAS ligand, and TNF-related apoptosis-inducing ligand (TRAIL), which bind to their corresponding receptors called ‘death receptors’^18^. FAS-associated death domain protein (FADD) is a critical adaptor protein for death receptor (DR)-mediated apoptosis which bridges the receptors (FAS, DR) with the downstream effector caspase-8 forming the death-inducing signaling complex (DISC) that ultimately leads to BID activation^19^. These sequences of events lead to release of caspase-activating factors, e.g., cytochrome-c, from mitochondria to induce apoptosis^20^.

In the last few decades, acquired knowledge of the molecular biology of colon cancer and its development in new therapeutic strategies has been steadily increasing^21^. Considering the poor responsiveness of colon cancer to conventional therapies, there has been need for anticancer drugs with high-efficacy and low-toxicity which might be beneficial for the elimination of tumors. Since years, considerable attention has been focused on many naturally occurring dietary phytochemicals. Crocetin (8, 8′‐diapocarotene‐8, 8′‐dioic acid), a major ingredient of saffron, from the flower of *Crocus sativus* L, is an important dietary ingredient. Growth inhibitory or pro-apoptotic properties of crocetin are reported in several malignant cells including pancreatic and breast cancer cells^22^,^23^. In addition, crocetin also inhibits TPA-induced skin tumors *in vivo*^24^ and exhibits protective effect against Benzo(a)pyrene-induced lung carcinogenesis^25^. However, the therapeutic role of crocetin in colorectal cancer with various p53 status requires detailed investigation. In this study, we have shown that crocetin induces p53-mediated cell death in functional p53-expressing cancer cells through BAX and PIDD-caspase-2-t-BID pathway. Whereas, in p53-impaired cancer cells, crocetin exploits p73-mediated FAS-FADD-caspase-8 activation and BID cleavage that ultimately brings its demise.

## Results

### Crocetin induces apoptosis in colon cancer cells independent of its p53 status

**Figure 1:**
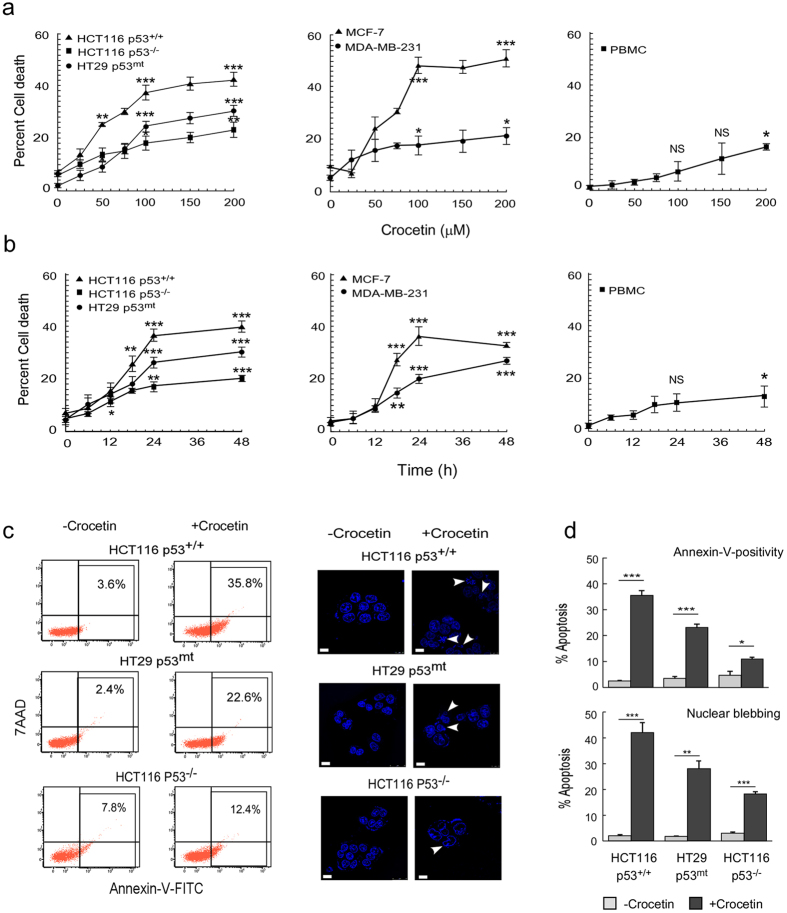
Crocetin induces apoptosis in colon cancer cells independent of its p53 status. (**a**) Dose-dependent effect of crocetin on percent death in HCT116 (p53^+/+^), HCT116 (p53^−/−^), HT29 (p53^mt^), MCF-7 (p53^+/+^), MDA-MB-231 (p53^mt^) cells and peripheral blood mononuclear (PBMC) cells was scored by trypan blue dye-exclusion method. (**b**) Time-dependent effect of crocetin (100 μM) on percent death in the above cells was determined. (**c**) Crocetin-treated (24 h) HCT116 (p53^+/+^) cells, HT29 (p53^mt^) cells and HCT116 (p53^−/−^) cells were flow cytometrically analyzed for Annexin-V-FITC- and 7AAD-positivity, regarded as apoptotic cells (*left*). Nuclear fragmentation or nuclear membrane blebbing was observed by DAPI-staining (as indicated by arrowheads) after 24 h of crocetin treatment in HCT116 (p53^+/+^ or p53^−/−^) and HT29 (p53^mt^) cells (*right*); scale bar represents 10 μm; magnification 40x. (**d**) Percent apoptosis obtained from Annexin-V-FITC- and 7AAD-positivity (*upper)* and DAPI staining (*lower)* in crocetin treated HCT116 (p53^+/+^ or p53^−/−^) and HT29 (p53^mt^) cells were represented graphically. Cells were analysed from three independent sets in each case. Values are mean ± SEM of three independent experiments in each case or representative of typical experiment *p < 0.05, **p < 0.01, ***p < 0.001.

A battery of wild-type, mutated p53-expressing or p53-null human cancer cells from different origins e.g., colon cancer (HCT116: p53^+/+^, p53^−/−^ and HT29: p53^mt^), breast cancer (MCF-7: p53^+/+^ and MDA-MB-231: p53^mt^) were selected to study the effect of crocetin. Crocetin showed various degrees of cell death in these functional p53-expressing and p53-deficient cancer cells with a preferred propensity towards p53-expressing cells (Fig. 1a,b). The effective doses of crocetin showed none or negligible toxicity to peripheral monocytes (Fig. 1a,b, *right*) indicating that the cytotoxic effect is specific for malignant cells. Annexin-V-positivity (Fig. 1c, *left*) and nuclear fragmentation or blebbing (Fig. 1c, *right*) data showed that corcetin (100 μM, 24 h) induced apoptosis in both the functional p53-expressing and functional p53-deficient cells. Interestingly, p53-null HCT116 cells also showed a significant number of apoptotic cells (Fig. 1c,d). These results together suggested that even in absence of functional p53 crocetin induces cancer cell death.

### p53 activation is crucial for functional p53-expressing HCT116 cell apoptosis

**Figure 2:**
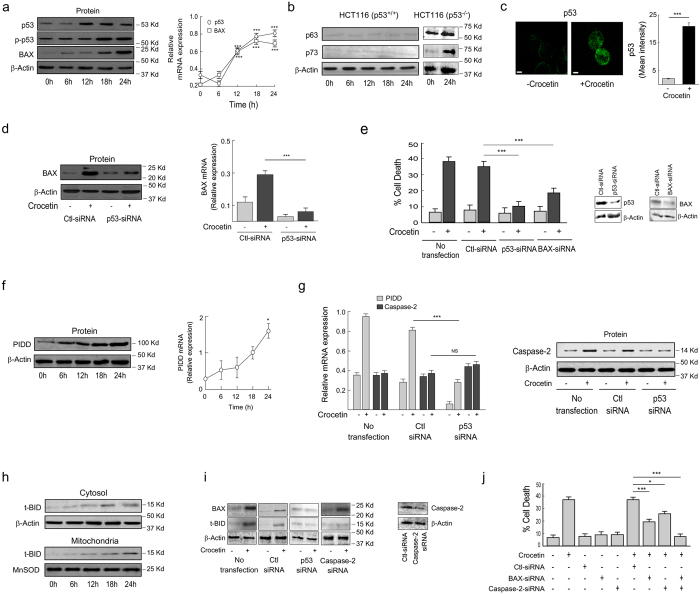
Crocetin induced p53-dependent apoptosis requires BAX induction and PIDD-mediated caspase-2 activation. (**a**) Wild-type p53-expressing HCT116 cells treated with crocetin (100 μM) at different time points were subjected to Western blot (*left*) and qPCR (*right*) analysis for the determination of p53 and BAX at protein, mRNA levels and phospho-p53at protein level. (**b**) Western blot analysis of p63 and p73 induction in crocetin-treated HCT116p53^+/+^ cells (*left*) and HCT116p53^−/−^ cells (*right*). (**c**) p53 induction in crocetin-exposed HCT116 cells was visualized by confocal microscopy (left) using Cy2-conjugated anti-p53 antibody (scale bar represents 10 μm; magnification 100X). Bar diagram represents the mean fluorescent intensity of p53 in crocetin-treated HCT116 cells (*right*). 10 cells were analysed from three independent experiments. (**d**) In p53-silenced crocetin-treated HCT116 cells BAX at protein (*left*) and mRNA levels (*right*) were determined by Western blot and qPCR. (**e**) In p53- and BAX-ablated crocetin-treated HCT116 cells (24 h, 100 μM), percent cell death was determined by Annexin-V-FITC-and 7AAD-positivity. Inset represents the immunoblot analysis to show transfection efficiency of p53- and Bax-siRNA. (**f**) Relative levels of PIDD at Protein (*left*) and mRNA (*right*) were determined in 100 μM crocetin-exposed HCT116 cells. (**g**) Relative levels of PIDD and caspase-2 mRNAs (*left*) and active form of caspase-2 (*right*) were determined in p53-silenced HCT116 cells in presence or absence of crocetin. (**h**) Time-dependent t-BID translocation from cytosol to mitochondria was determined by Western blot in presence of 100 μM crocetin. (**i**) BAX induction and t-BID formation were determined in crocetin-treated p53- and caspase-2-silenced HCT116 cells by Western blot analysis (*left*). Transfection efficiency of caspase-2 siRNA was analyzed by immunoblot. (**j**) Percent cell death in crocetin-treated BAX- and caspase-2-silenced HCT116 cells was determined by Annexin-V-FITC- and 7AAD-positivity (24 h, 100 μM). β-actin and MnSOD were used as internal loading control. Values are mean ± SEM of three independent experiments in each case or representative of typical experiment *p < 0.05, **p < 0.01, ***p < 0.001.

Since crocetin induced apoptosis irrespective of the p53 status with a preferred propensity towards p53-expressing cells, we wanted to check whether p53 has any role on such cell death or not. To that end, we observed that crocetin augmented p53 at protein level with simultaneous induction of p53-Ser-phosphorylation and concomitant BAX induction (Fig. 2a, *left*; Supple. Fig. 1) in wild-type p53-expressing HCT116 cells. The levels of p53 and BAX-mRNA were also elevated due to crocetin exposure (Fig. 2a, *right*). This phytochemical did not change the status of the two structurally and functionally related p53-paralogues p63 and p73 (Fig. 2b, *left*), which can also promote apoptosis either in parallel or in concert with p53^26^,^27^. Our confocal microscopic data further supports the amplification data of p53 due to crocetin exposure (Fig. 2c). The role of p53 in BAX transactivation and their involvement in crocetin-induced apoptosis was further confirmed by p53-RNA-interferences (Fig. 2d,e). Silencing of BAX revealed partial reduction of apoptosis in contrast to that of p53 (Fig. 2e). This raised the possibility of existence of parallel death pathway other than p53-BAX axis. These results establish the contribution of BAX in crocetin-induced apoptosis in p53-dependent manner.

### In addition to BAX, PIDD activation is important for functional p53-expressing cell apoptosis

Previous results prompted us to study the contribution of parallel p53-dependent pathway in crocetin-induced apoptosis. Among the well-known p53-target genes, PIDD is a major one that links additional components of p53-apoptosis pathway. Caspase-2, a well-recognized mediator of apoptosis, is a component of a large complex PIDDosome (formed by PIDD and the adaptor protein RAIDD), is likely to regulate crocetin-induced apoptosis^11^. Our investigations revealed an elevated amount of PIDD at the transcriptional and translational levels in crocetin-exposed cells (Fig. 2f). Moreover, p53-silencing completely blocked crocetin-induced PIDD transcription implicating the involvement of p53-mediated PIDD transactivation (Fig. 2g, *left*). Interestingly, p53 ablation didn’t alter caspase-2 at mRNA level but completely blocked its activation which was evident from the Western blot intensities for active form of caspase-2 (Fig. 2g, *right*). All these results together suggested the role of p53-transactivated PIDD and BAX in crocetin-induced apoptosis.

Since, caspase-2 is known to cleave BID to generate active t-BID^14^; we analyzed the translocation of t-BID from cytosol to mitochondria. Our Western blot data suggested gradual accumulation of t‐BID in the cytosolic fraction with its concomitant translocation to the mitochondria upon crocetin‐treatment (Fig. 2h). To validate our hypothesis, a series of RNA-interference studies were done. Silencing p53 and caspase-2 individually completely blocked crocetin-induced t-BID formation (Fig. 2i). On the contrary, BAX augmentation was significantly reduced upon p53-silencing but remained unchanged when caspase-2 was ablated (Fig. 2i). Finally, to establish the contribution of both BAX and caspase-2 together in p53-dependent death pathways, BAX and caspase-2 were ablated alone or in combination using specific siRNAs. It was observed that manipulating BAX or caspase-2 individually reversed crocetin-induced cell death partially, whereas co-transfection of BAX- and caspase-2-siRNA completely blocked crocetin-induced apoptosis (Fig. 2j). The above findings implicate p53-transactivated BAX and PIDD-mediated caspase-2 activation in the apoptosis of functional p53-expressing cancer cells.

### Crocetin-induced HCT116 p53^+/+^ cell apoptosis requires mitochondrial transmembrane potential loss and activation of caspases

**Figure 3:**
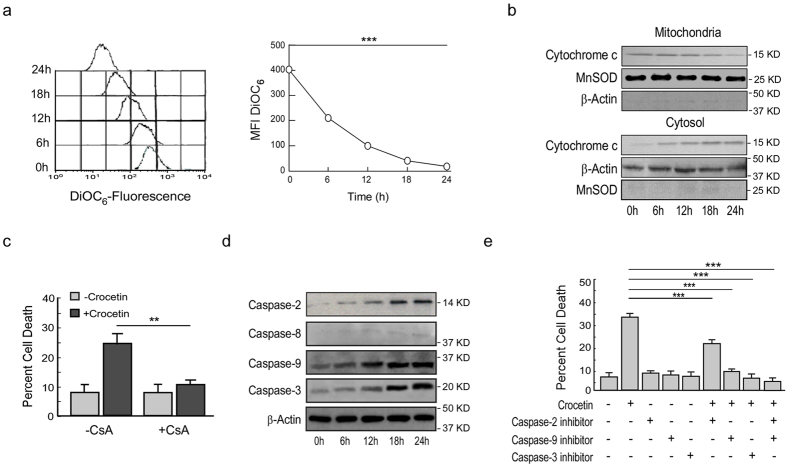
Crocetin-induced functional p53-expressing cell apoptosis requires mitochondrial transmembrane potential loss and activation of caspases. (**a**) Crocetin-exposed (100 μM) HCT116 cells were flow cytomtrically assessed for MTP loss by DiOC_6_-fluorescence (*left*) and mean fluorescence intensities were represented graphically (*right*). (**b**) Crocetin-treated (100 μM) HCT116 cells were analyzed for cytochrome-c release from mitochondria to cytosol by Western blotting. (**c**) Percent CsA-primed crocetin-treated HT29 cell death (100 μM) was determined by Annexin-V-FITC- and 7AAD-positivity at 24 h. (**d**) The levels of active form of caspases (-2, -8, -9 and -3) in 100 μM crocetin-exposed HCT116 cells were determined by Western blotting. (**e**) Caspase-2, -9 and -3 inhibitor-primed (20 μM) HCT116 cells were exposed to crocetin for 24 h and percent cell death was scored by Annexin-V-FITC- and 7AAD-positivity. β-actin and MnSOD were used as internal loading control. Values are mean ± SEM of three independent experiments in each case or representative of typical experiment. *p < 0.05, **p < 0.01, ***p < 0.001.

BAX and t-BID data prompted us to evaluate the involvement of mitochondria in crocetin-induced functional p53-expressing cell apoptosis. Decrease in DiOC_6_-fluorescence (Fig. 3a) and increase in cytochrome-c release from mitochondria (Fig. 3b) indicated that crocetin damaged mitochondrial transmembrane potential (MTP) in these cells. Cyclosporine-A (CsA), the inhibitor of mitochondrial pore formation, significantly reduced the crocetin-induced apoptosis indicating that mitochondrial disruption is the reason for such death (Fig. 3c). Increase in active forms of caspase-2, -9 and -3, but not caspase-8, indicated the involvement of initiator and executioner caspases in p53-dependent intrinsic apoptotic pathway (Fig. 3d). Dependence of caspases in p53-mediated apoptosis was finally established using cell-permeable inhibitors of caspases (caspase-2: Z-VDVAD-FMK, caspase-9: Z-LEHD-FMK and caspase-3: Z-DEVD-FMK) individually or in combination followed by crocetin-treatment. Our data showed a partial reduction of cell death in caspase-2 inhibited condition, whereas, caspase-9 or caspase-3 inhibition caused complete reversal of the effect (Fig. 3e). Together, all these data suggested that crocetin promotes apoptosis of HCT116p53^+/+^ cells *via* mitochondrial disruption through activation of caspase-cascade.

### Crocetin induces apoptosis of mutant-p53-expressing cells through p73-mediated FAS activation

**Figure 4:**
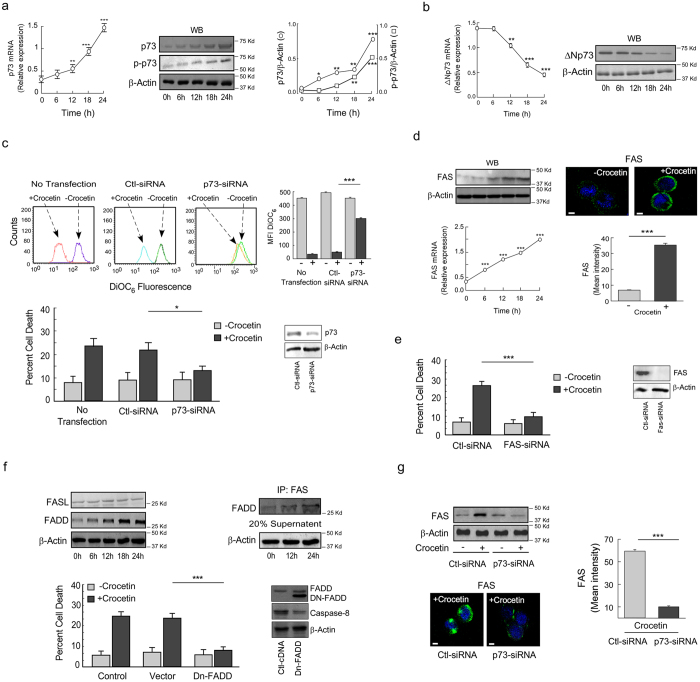
Crocetin induces mutant-p53-expressing cell apoptosis through p73-mediated FAS activation. Mutant-p53-expressing HT29 cells were treated with 100 μM crocetin for different time intervals and the relative levels of p73 at mRNA (*left*), protein and phosphoprotein (*middle*) were analyzed by qPCR and immunoblotting. Quantification and normalization of p73 and p-p73 to total actin (*right*). (**b**) Relative levels of ∆Np73 at mRNA (*left*) and protein (*right*) in 100 μM crocetin-treated HT29 cells were analyzed by qPCR and immunoblotting. (**c**) p73-silenced HT29 cells were treated with 100 μM crocetin and the MTP loss was assessed flow cytometrically at 24 h and represented graphically (*upper)*. In parallel, percent cell death was scored by Annexin-V-FITC- and 7AAD-positivity (*lower*). Inset represents the transfection efficiency of p73-siRNA. (**d**) Crocetin-induced FAS induction was determined at protein or mRNA levels by immunoblot (*upper left*) and qPCR (*lower left*). FAS aggregation at surface of crocetin-treated cells was determined by confocal microscopy using FITC-tagged anti-FAS antibody (*upper right*); scale bar represents 10 μm; magnification 100x. Bar diagram represents the mean fluorescence intensity of FAS in crocetin-treated HT29 cells (*lower right*). 10 cells were analyzed from each of three independent experiments. (**e**) Crocetin-treated FAS-silenced HT29 cells were used to analyze percent cell death by Annexin-V-FITC- and 7AAD-positivity at 24 h. Inset represents the transfection efficiency of FAS-siRNA. (**f**) Expression level of FASL and FADD were determined by Western blotting (*upper left*). FAS-associated FADD was detected by co-immunoprecipitation (with anti-FAS antibody) and Western blotting with anti-FADD antibody (*upper-right*). Dn-FADD-transfected HT29 cells were treated with crocetin and percent cell death was scored by Annexin-V-FITC- and 7AAD-positivity (*lower*). Inset represents the immunoblot analysis for transfection efficiency and effector function of Dn-FADD. (**g**) p73-silenced HT29 cells were treated with crocetin for 24 h and the level of FAS was analyzed by immunoblot and confocal microscopy (*left*). Scale bar represents 10 μm; magnification 100x. Bar diagram represents the mean fluorescent intensity of FAS in these cells (*right*). 10 cells were analyzed from each of three independent experiments. β-actin was used as internal loading control. Values are mean ± SEM of three independent experiments in each case or representative of typical experiment. *p < 0.05, **p < 0.01, ***p < 0.001.

Results of Fig. 1 indicated that crocetin induced apoptosis irrespective of the functional status of p53, which led us to explore the existence of p53-independent apoptosis in functional p53-deficient cells. To that end, we observed that crocetin augmented p73 both at mRNA as well as at protein level in p53-mutant HT29 cells and simultaneously induced p73-Ser-phosphorylation (Fig. 4a; Supple. Fig. 1). Since, endogenous ∆Np73 is a dominant negative inhibitor of p73^28^, next, we investigated the effect of crocetin on the status of ∆Np73. Our data showed that crocetin efficiently blocked ∆Np73 expression both at transcript as well as at protein levels (Fig. 4b), indicating that in functional p53-deficient condition, it exploits p73 to induce apoptosis. As validation of these assumptions experiments using p73-siRNA showed significant impediment of crocetin-induced cell death *via* decreasing DiOC_6_-fluorescence (Fig. 4c). Above findings implied crucial role of p73 in the induction of apoptosis in functional p53-impaired cells.

Involvement of p73 prompted us to examine the effect of crocetin on FAS-mediated apoptosis in these p53-impaired cells, since it is known that p73 transactivates FAS^29^. To that end, we observed that crocetin augmented FAS both at mRNA and protein levels (Fig. 4d, *left*) and increased FAS clustering in the cell membrane (Fig. 4d, *right*). FAS ablation completely blocked cell death indicating its involvement in apoptotic pathway (Fig. 4e). Interestingly, we noticed that the basal level of FAS ligand is maintained throughout the incubation period (Fig. 4f, *left*) which might be sufficient in killing of cells. The level of FADD increased dramatically and was associated with FAS upon crocetin exposure (Fig. 4f, *upper*). In parallel, upon transfection of dominant-negative FADD clone (Dn‐FADD), we witnessed an inhibition of apoptosis (Fig. 4f, *lower*) which validated the role of FAS-FADD in crocetin-induced apoptosis in functional p53-deficient cancer cells. Next, to draw a link between FAS and p73, we silenced p73 in HT29p53^mt^ cells, which revealed a significant reduction of FAS as well as its clustering in the membrane (Fig. 4g). All the above findings indicate that in absence of functional p53, p73 induces HT29 cell death *via* FAS-FADD death-receptor pathway.

### p73-mediated apoptosis of functional p53-deficient cancer cells requires caspase-8 activation

**Figure 5:**
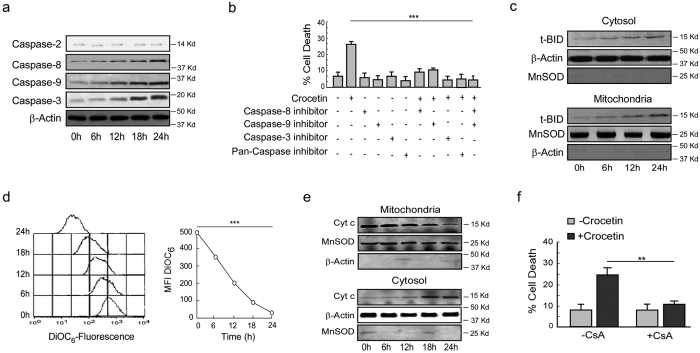
p73-mediated functional p53-deficient cancer cell apoptosis requires caspase-8 activation. (**a**) Expression patterns of active form of caspase-2, -8, -9 and -3 in 100 μM crocetin-exposed HT29 cells were determined by Western blotting. (**b**) Caspase-8, -9 and -3 inhibitor-primed (20 μM) HT29 cells were exposed to 100 μM crocetin for 24 h and percent cell death was scored by Annexin-V-FITC- and 7AAD-positivity.(**c**) Translocation of t-BID from cytosol to mitochondria in crocetin-treated HT29 cells was determined by Western blotting. (**d**) HT29 cells treated with 100 μM crocetin were flow cytomtrically assessed for MTP loss at 24 h by DiOC_6_-fluorescence (*left*) and represented graphically (*right*). (**e**) Cytochrome-c release from mitochondria to cytosol was analyzed by Western blotting. (**f**) Graphical representation of percent CsA-primed crocetin-treated HT29 cell death was determined by Annexin-V-FITC- and 7AAD-positivity at 24 h. β-actin and MnSOD were used as internal loading control. Values are mean ± SEM of three independent experiments in each case or representative of typical experiment.

Since caspase-8 is known to be activated in receptor-mediated death-pathway^30^, we intended to study the effect of crocetin on the activation of this caspase cascade in functional p53-deficient HT29 cells. It was observed that crocetin activates caspase-8, -9 and -3 (Fig. 5a), but not caspase-2 (Fig. 5a), thereby implicating both initiator and executioner caspases in such death process. Studies with individual caspase inhibitor or pan-caspase inhibitor abrogated crocetin-induced cell death (Fig. 5b) thereby validating that crocetin exerts its apoptogenic effect through caspase-8-induced caspase-9 and -3-dependent manner. In addition, we also observed the accumulation of t-BID in mitochondria (Fig. 5c) which is known to be achieved through caspase-8-dependent pathway. Finally, we witnessed the participation of mitochondria through the transmembrane-permeability loss (Fig. 5d) and cytochrome-c release (Fig. 5e) as well as abrogation of cell death by cyclosporine-A (Fig. 5f).

**Figure 6:**
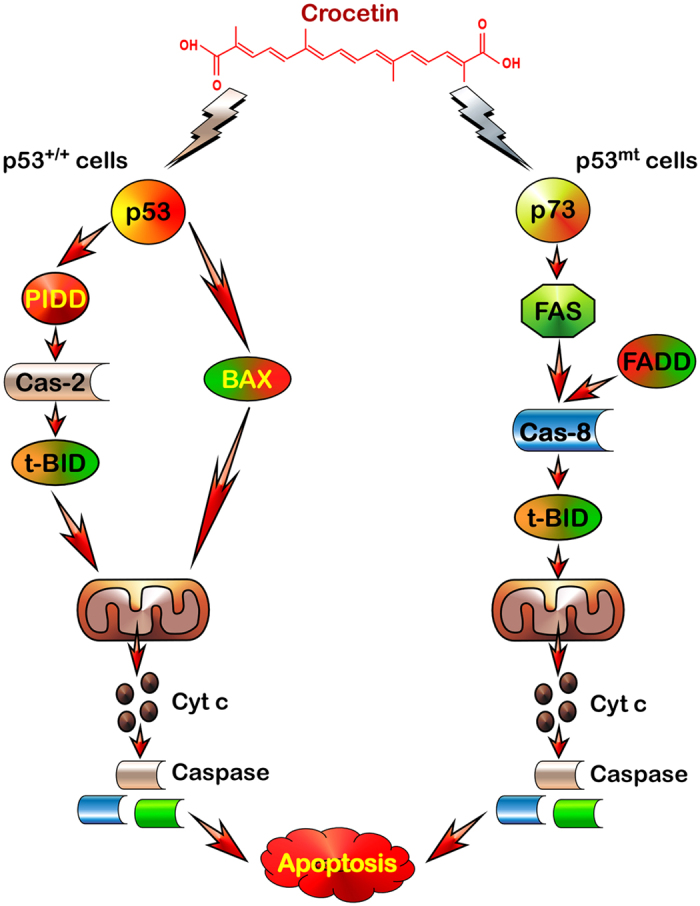
Schematic diagram representing mechanism of p53-dependent and p53-independent death of wild-type (p53^+/+^) and mutant p53-expressing (p53^mt^) colon cancer cells as a result of crocetin treatment.

In summary, it is evident that in functional p53-expressing cancer cells crocetin exploits p53-induced PIDD and caspase-2 to activate BID and BAX to execute intrinsic apoptotic signalling. On the other hand in functional p53-deficient cells, p53-paralogue p73 transactivates FAS to induce FAS-FADD-caspase-8-t-BID axis for activating mitochondrial-death pathway (Fig. 6).

## Discussion

In half of all colorectal cancers, high-incidence of p53 (TP53) gene mutations^31^ makes it a major cause of mortality throughout the world. Tumor suppressor p53 controls a broad range of cellular responses comprising of induction of a transient (cell cycle arrest) or a permanent (senescence) block of cellular proliferation, or activation of apoptosis in response to DNA-damage in cells^32^. Shutdown of these normal developmental processes requires inactivation of p53 to develop tumorigenesis. From an evolutionary perception, besides having a firm regulation, activities of p53 must be carried out by its counterparts in a p53-impaired system. Accordingly, though the master regulator p53 is known to be an effective target for therapeutic studies, ongoing researches also highlight the anti-tumorigenic actions displayed by its structural analogue p73 in many human cancers. Such an in-build balance favors an environment for killing of the immortal cancer cells even in absence of a tumor suppressor protein. To initiate the same type of cellular responses without toxicity as known to be associated with the synthetic drugs, many natural compounds are suggested to be used as potential chemopreventive and chemotherapeutic agents^33^.

Here, we emphasized upon two different aspects of crocetin-induced apoptotic cell death in colon cancer, namely, p53-dependent and p53-independent ones. Crocetin, a promising candidate for cancer chemoprevention^34^, has been known for restoring vincristine-sensitivity in vincristine-resistant breast cancer cell MCF-7/VCR^35^. Our study highlights the crucial role of crocetin in inducing apoptosis in a series of cancer cells, irrespective of their p53 status. To uncover the p53-dependent way of cell death, we chose HCT116 (p53^+/+^) cells as our model. Among various critical apoptosis-controlling genes, p53-transactivated BAX has been the central topic of discussion^36^,^37^. Our evidences also suggested the pro-apoptotic function of BAX in crocetin-induced functional p53-expressing cell death.

Although, saffron extract has shown its pro-apoptotic and anti-inflammatory effects against some cancer cells^38^, its contribution in apoptotic processes has yet to be explored in detail in colorectal cancer. PIDD, a cytosolic protein, is one of the transcriptional targets of p53 which is known to function as a p53 mediator of apoptosis. Previous studies suggested that over-expression of PIDD inhibits cell growth in a p53-like manner^15^. It has been shown that antisense inhibition of PIDD attenuates apoptosis in response to DNA-damage-induced p53 activation^39^ and PIDD activates caspase-2^11^,^40^. Here we report that p53-transactivated PIDD activates caspase-2 and sensitizes cancer cells to apoptosis in response to crocetin. Activated caspase-2 has been shown to cleave BID causing the mitochondrial accumulation of t-BID. Our findings for the first time highlight the potential action of crocetin in inducing functional p53-expressing cell apoptosis in which p53 transactivates BAX and PIDD, both of which converge into mitochondria to cause MTP loss, cytochrome-c release from the mitochondrial inter-membrane space, and increase activation of initiator and executioner caspases thereby culminating in apoptosis^41^.

Since p73, unlike p53, is rarely mutated in human cancer^42^, we proposed that anti-cancer agent which can activate p73 can be a potential therapeutic tool against mutant p53- bearing cancers. Previously, our lab reported that in p53-impaired cancer cells, DNA-damage caused accumulation of p53-paralogue p73 *via* Chk-1 that strongly impacted BAX induction and p53-independent apoptosis^36^. Here we observed an alternative mechanism of induction of apoptosis through p73 in functional p53-deficient colon cancer cells. To that end, we evidenced that crocetin up-regulated p73 and down-regulated ∆Np73 thereby disabling the dominant negative effect of ∆Np73 on p73 in p53-impaired cancer cells. Reports suggest that various plant polyphenols induce and activate FAS receptor in cancer cells^43^,^44^,^45^,^46^. It is also reported that in p53-deficient cells p73 regulates FAS induction^47^. In line with these evidences, we observed that in p53-impaired cells, crocetin induced FAS, increased their clustering on the cell surface and caused FAS-FADD association in p73-dependent manner. This deadly-association ultimately activated BID through caspase-8 to alter mitochondrial transmembrane potential and activated executioner caspase cascade. Hence, p73 bypassed the drawbacks of mutated p53 by up-regulating FAS/FADD death receptor pathway in presence of crocetin.

In gist, our results evident different apoptotic mechanisms in response to crocetin in colon cancer cells with various p53 statuses. In functional p53-expressing cancer cells, crocetin targets p53-mediated BAX activation in one hand and PIDD/caspase-2-mediated BID activation on the other which together converge to mitochondria to cause transmembrane permeability loss and caspase cascade activation. However, functional p53-deficient colon cancer cells utilize an alternative p73-FAS-FADD-caspase-8 axis that culminates into mitochondrial disruption and apoptosis. Since, functions of p53 is commonly lost in many cancers, the newly suggested p53-indpendent mechanism can be allotted to its homologue p73, which may prove to be of potential therapeutic significance for designing novel strategies to improve efficacy in colon cancer treatment.

## Materials and Methods

### Cell culture and treatments

Colon cancer cell lines HCT116 (p53^+/+^), HCT116 (p53^−/−^) and HT29 (p53^mt^), breast cancer cell lines MDA-MB-231 (p53^mt^) and MCF-7 (p53^+/+^) were obtained from NCCS, India. Blood collected from healthy human volunteers with informed consent (Institutional Review Board 1382) was centrifuged over Ficoll-Hypaque density gradient (Amersham Pharmacia, Uppsala, Sweden) to obtain total peripheral blood mononuclear cells (PBMC)^48^. Cells were routinely maintained in complete DMEM/RPMI 1640 at 37 °C in a humidified incubator containing 5% CO_2_. Cells were allowed to reach confluency before use. Dose-dependent and time-dependent cell death experiments were performed using Trypan blue-exclusion test^49^. All experiments were performed in accordance with approved guidelines and regulations of the Helsinki Declaration (http://www.wma.net/en/30publications/10policies/b3/index.html). All experimental protocols were approved by informed consent (IRB-1382), Human Ethics Committee, Bose Institute (Approval No: BIHEC/2010-11/2).

### Treatment of cells

Cells were treated with different doses of crocetin (MP Biomedical, India) at different time intervals to select the optimum dose (100 μm) and time (24 h) for cancer cell death which was used in all experiments unless stated otherwise. HCT116 and HT29 cells were pre-treated with 20 μM each of the specific caspase-2 (Z-VDVAD-FMK), caspase-8 (Z-IETD-FMK), caspase-9 (Z-LEHD-FMK), caspase-3 (Z-DEVD-FMK) and Pan-caspase (Z-VAD-FMK) inhibitor for 2 h, (Calbiochem, ED chemicals, NJ) or for 1 h with 25 μM mitochondrial pore blocker CsA (Merck, Germany) prior to treatment with crocetin.

### Flow cytometry

For the determination of apoptotic cell death, cells were stained with 7AAD and Annexin-V-FITC according to the protocol (BD Pharmingen) and analyzed in flow cytometer (FACS Aria, BD). Electronic compensation of the instrument was done to exclude overlapping of the emission spectra. Total 10,000 events were acquired for analysis using CellQuest software (Becton Dickinson, San Jose, CA). For the assessment of MTP, cells were incubated with crocetin for 24 h and then for an additional 15 min with 40 nM DiOC_6_ at 37 °C in dark. Cells were analyzed flow cytometrically for DiOC_6_-fluorescence using logarithmic amplification by CellQuest software.

### Fluorescence imaging

Chromatin condensation and nuclear fragmentation was analyzed microscopically using standard protocol. Briefly, cells were grown on cover slips, fixed with 4% para-formaldehyde, permeabilized with 0.1% Triton-X-100 and incubated with 4, 6-diamidino-2-phenylindole (DAPI; BD Pharmingen, CA). Finally, coverslips were mounted using DPX mounting medium. Morphology of the cell nuclei was subsequently visualized using a fluorescence microscope to ascertain apoptotic cell death (Leitz microscope fitted with epifluorescence illuminator, Carl Zeiss, Germany). To study protein expression using fluorescence imaging, cells grown on cover slips were fixed with 4% para-formaldehyde, permeabilized with 0.1% TritonX-100 and were stained with 1:100 dilution of anti-p53, anti-p73 and anti-FAS antibody (Santa Cruz, CA), followed by staining with 1:100 dilution of Cy2-conjugated secondary antibody (Jackson ImmunoResearch Laboratory, PA) and visualized with confocal microscope (Carl Zeiss, Germany). Images of DAPI staining were captured at 40x magnification whereas others were represented at 100x.

### Co-immunoprecipitation and immunoblotting

For whole cell lysates, cells were homogenized in buffer (20 mM Hepes, pH 7.5, 10 mM KCl, 1.5 mM MgCl_2_, 1 mM Na-EDTA, 1 mM Na-EGTA, and 1 mM DTT) supplemented with protease and phosphatase cocktail inhibitor. Mitochondrial and cytosolic fractions were prepared according to Lahiry *et al*.^50^. For direct western blot analysis, a total of 50 μg of protein was resolved using SDS-PAGE and transferred to nitrocellulose membrane and probed with specific antibodies like, anti-p53 (DO-1, mAb), p-p53 (pAb), BAX (pAb), p63 (mAb), p-p73 (pAb), ΔNp73 (mAb), PIDD (pAb), caspase-3 (pAb), caspase-9 (mAb), caspase-8 (mAb), t-BID (pAb), cytochrome-c (pAb), FAS (pAb), FASL (pAb), FADD (pAb) (Santa Cruz, CA, USA), anti-p73 (mAb), caspase-2 (mAb) (Cell Signaling, MA, USA) and visualized by chemiluminescence (GE Biosciences, NJ, USA). For the determination of direct interaction between two proteins, co-immunoprecipitation technique was employed^51^. For co-immunoprecipitation of FAS and FADD, FAS immunocomplex from whole cell lysate was purified using anti-FAS antibody and protein A-Sepharose beads (Invitrogen, Carlsbad, USA). The immunopurified protein was immunoblotted with anti-FADD antibody. Equal protein loading was confirmed with anti-β-actin and anti - MnSOD antibody (mAb) (Santa Cruz, CA, USA).

### Real-time-PCR

Two μg of the total RNA was extracted from cells using TRIzol Q16 reagent (Invitrogen, Carlsbad). The mRNA expression levels were measured by quantitative real-time PCR using a QuantiTect SYBR^R^ Green real-time-PCR kit and the iCycler real-time detection system and software according to the manufacturer’s instructions. Passive reference dye (ROX) was used to normalize the SYBR Green/double-stranded DNA complex signal during analysis to correct for well-to-well variation and sampling loading error. Amplification products using SYBR Green detection were checked using melting curve with ICYCLER software (version-3; Bio-Rad, Hercules, CA) and by 1% agarose gel electrophoresis to confirm the size of the DNA fragment and that single product was formed. Samples were compared using the relative (comparative) Ct method. The Ct value, which is inversely proportional to the initial template copy number, is the calculated cycle number where the fluorescence signal emitted is significantly above background levels. Expression level of the housekeeping gene, GAPDH, was used to normalize for variations in amount of RNA and RNA purity. The fold induction or repression by real-time RT-PCR was calculated according to the following formula: fold change =2^−∆∆ct^; where ∆∆ct = ∆ct control − ∆ct treatment, and ∆ct = target gene ct - GAPDH ct. Primers sequences used in real-time are: BAX (5′-TTTGCTTCAGGGTTTCATCC-3′ and 5′-CAGTTGAAGTTGCCGTCAGA-3′), p53 (5′-GGCCCACTTCACCGTACTAA-3′ and 5′-GTGGTTTCAAGGCCAGATGT-3′), p73 (5′-CAGACAGCACCTACTTCGACCTT-3′ and 5′-CCGCCCACCACCTCATTA-3′), ΔNp73 (5′-TTCAGCCAGTTGACAGAACTAAGG-3′ and 5′-GCGTTTGTTGGCATTT-3′), FAS (5′-CAAGGGATTGGAATTGAGGA-3′ and 5′-GACAAAGCCACCCCAAGTTA-3′), PIDD (5′-ACTTCTCCTGGTACTGGCTCTG-3′ and 5′-AAGGCT GCAAAGAACTTCTCAC-3′), and GAPDH (internal control): (5′-CAGAACATCATCCCTGCCTCT-3′ and 5′-GCT TGACAAAGTGGTCGTTGAG-3′).

### Plasmids, siRNA and transfections

PcDNA3 vector containing dominant-negative (Dn)-FADD (Biotechnomics Lab, India) and control pcDNA3.0 vector were separately introduced into HT29 cells using Lipofectamine 2000 (Invitrogen, Carlsbad, CA) according to the protocol provided by the manufacturer. Stably expressing clones were isolated by limiting dilution and selection with 1 mg/ml G418 sulfate (Cellgro) and G418 resistant cells were cloned and screened by immunoflourescence or western blotting with specific antibodies. For endogenous silencing of specific genes, cells were transfected with 300 pmole of control-, p53-, p73-, BAX-, FAS- or caspase-2-siRNA (Santa Cruz, CA) and lipofectamine-2000 separately for 12 h. The mRNA and protein levels were determined by qPCR and Western blotting respectively.

### Statistical analyses

Values are shown as standard error of mean (SEM) except otherwise indicated. Comparison of multiple experimental groups was performed by 2-way ANOVA followed by a post-hoc Bonferroni modification of multiple comparison t-tests. Data were analyzed and when appropriate, significances of the differences between mean values were determined by Student’s t test. Results were considered significant at p ≤ 0.05.

## Additional Information

**How to cite this article**: Ray, P. *et al*. Crocetin exploits p53-induced death domain (PIDD) and FAS-associated death domain (FADD) proteins to induce apoptosis in colorectal cancer. *Sci. Rep*. **6**, 32979; doi: 10.1038/srep32979 (2016).

## Supplementary Material

Supplementary Information
