# Supplementary material for: Crocetin exploits p53-induced death domain (PIDD) and FAS-associated death domain (FADD) proteins to induce apoptosis in colorectal cancer
Source: Sci Rep. 2016 Sep 13;6:32979. doi: 10.1038/srep32979 (PMC5020693; doi:10.1038/srep32979)
Supplement: Supplementary Information [file srep32979-s1.pdf]

Crocetin exploits p53-induced death domain (PIDD) and FAS-associated death domain (FADD) proteins to induce apoptosis in colorectal cancer

*Pallab Ray[ξ], Deblina Guha[ξ], Juni Chakraborty, Shuvomoy Banerjee, Arghya Adhikary, Samik Chakraborty, Tanya Das[1] and Gaurisankar Sa**

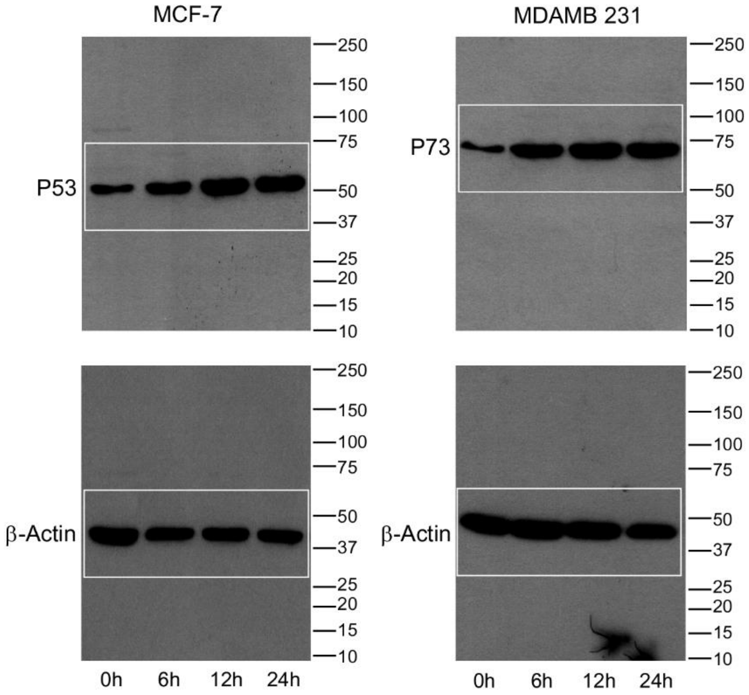

Supplementary Figure-1. Crocetin induces p53 and p73 in breast cancer cell lines bearing different p53 status. Wild-type p53-expressing MCF-7 cells and mutated p53-expressing MDAMB 231 cells treated with crocetin (100μM) at different time points were subjected to Western blot analysis for determination of status of p53 and p73 respectively.
